# Hes1 regulates embryonic stem cell differentiation by suppressing Notch signaling

**DOI:** 10.1111/j.1365-2443.2010.01413.x

**Published:** 2010-07

**Authors:** Taeko Kobayashi, Ryoichiro Kageyama

**Affiliations:** 1 Institute for Virus Research, Kyoto UniversityKyoto 606-8507, Japan; 2 Japan Science and Technology Agency, CRESTKyoto 606-8507, Japan

## Abstract

Embryonic stem (ES) cells display heterogeneous responses upon induction of differentiation. Recent analysis has shown that *Hes1* expression oscillates with a period of about 3–5 h in mouse ES cells and that this oscillating expression contributes to the heterogeneous responses: Hes1-high ES cells are prone to the mesodermal fate, while Hes1-low ES cells are prone to the neural fate. These outcomes of Hes1-high and Hes1-low ES cells are very similar to those of inactivation and activation of Notch signaling, respectively. These results suggest that Hes1 and Notch signaling lead to opposite outcomes in ES cell differentiation, although they work in the same direction in most other cell types. Here, we found that Hes1 acts as an inhibitor but not as an effector of Notch signaling in ES cell differentiation. Our results indicate that sustained *Hes1* expression delays the differentiation of ES cells and promotes the preference for the mesodermal rather than the neural fate by suppression of Notch signaling.

## Introduction

Notch signaling is known to regulate the maintenance of various types of stem cells (Artavanis-Tsakonas *et al.* 1999). By interaction with Notch ligands such as Deltalike1 (Dll1) and Jagged1 (Jag1), the transmembrane protein Notch is cleaved by γ-secretase, releasing Notch intracellular domain (NICD). NICD translocates into the nucleus, forms a complex with the DNA-binding protein RBPj and induces the expression of downstream effectors such as the transcriptional repressor genes *Hes1* and *Hes5* (Kageyama *et al.* 2007). Hes1 and Hes5 then repress expression of differentiation determination genes, thereby maintaining stem/progenitor cells. For example, in the developing nervous system, NICD leads to up-regulation of *Hes1* and *Hes5* and down-regulation of proneural genes such as *Mash1* and to maintenance of neural stem/progenitor cells; in the absence of both *Hes1* and *Hes5*, NICD is unable to maintain neural stem/progenitor cells, allowing premature neuronal differentiation (Ohtsuka *et al.* 1999). These results suggest that Notch signaling regulates the stem/progenitor cell state by inducing *Hes1* and *Hes5*.

Recent studies have revealed that Notch signaling is not always involved in maintenance of the stem/progenitor cell state. Both activation of Notch signaling by expression of NICD and inactivation of Notch signaling by deletion of *RBPj* do not affect the stem cell state of embryonic stem (ES) cells (Schroeder *et al.* 2003; Lowell *et al.* 2006; Noggle *et al.* 2006). However, under differentiation conditions, misexpression of NICD directs ES cells into neuroectodermal progenitor cells (Lowell *et al.* 2006), while inactivation of Notch signaling by treatment with γ-secretase inhibitors or by genetic inactivation of *Notch1* or *RBPj* promotes ES cell differentiation into cardiac mesodermal cells (Schroeder *et al.* 2003; Nemir *et al.* 2006; Jang *et al.* 2008). These results suggest that the activity of Notch signaling is important for the cell fate choice of ES cells rather than for the maintenance of the stem cell state (Noggle *et al.* 2006; Yu *et al.* 2008).

We have recently found that Hes1 is not involved in maintenance of the undifferentiated state in ES cells but is important for differentiation of these cells. Hes1 is expressed at variable levels by mouse ES cells under the control of leukemia inhibitory factor (LIF) and bone morphogenetic protein (BMP) but not of Notch signaling, and Hes1 expression oscillates with a period of about 3–5 h (Kobayashi *et al.* 2009). Interestingly, in ES cells, Hes1 expression levels at the time of induction of differentiation affect the preference in the cell fate choice: Hes1-high ES cells are prone to the mesodermal fate and Hes1-low ES cells are prone to the neural fate (Kobayashi *et al.* 2009). Furthermore, inactivation of *Hes1* facilitates neural differentiation of ES cells more uniformly. The effect caused by inactivation of *Hes1* is different from the one caused by inactivation of Notch signaling in ES cells. Inactivation of Notch signaling preferentially induces mesodermal differentiation, or rather the same as the one caused by induction of Hes1, although Hes1 and Notch have the same effects in most other cell types (Kageyama *et al.* 2007).

In this study, to understand the mechanism of how Hes1 regulates ES cell differentiation, we analyzed ES cells with *Hes1* cDNA knocked-in into the Rosa26 locus, which express Hes1 in a sustained manner (Kobayashi *et al.* 2009). These ES cells were delayed in differentiation but then differentiated into the mesodermal progenitor cells more preferentially than the wild-type ES cells, although Hes1 is expressed by the progenitor cells of all three germ layers (Sasai *et al.* 1992; Jensen *et al.* 2000). We further found that Hes1 does not mimic but antagonizes Notch signaling by directly repressing the expression of Notch ligands. These results suggest that Hes1 regulates the fate choice of ES cell differentiation by suppressing the Notch signaling.

## Results

### Sustained Hes1 expression delays differentiation of ES cells

**Figure 1:**
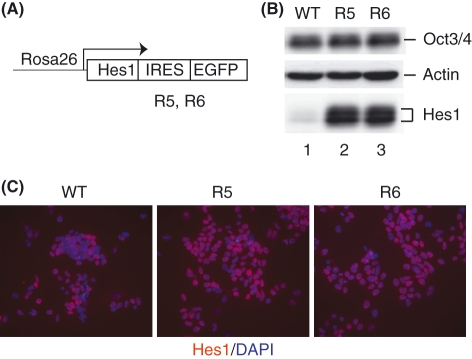
Hes1 protein expression in Hes1-sustained embryonic stem (ES) cells. (A) The structure for sustained Hes1 expression. The *Hes1* cDNA with the IRES-EGFP sequence was knocked-in into the Rosa26 locus, so that Hes1 and EGFP were constitutively expressed from the Rosa26 promoter (Kobayashi *et al.* 2009). (B) Hes1 and Oct3/4 expression in the wild-type (WT) and Hes1-sustained (R5, R6) ES cell lines. Actin is a loading control. (C) Immunostaining of Hes1 (red) with DAPI staining (blue) in ES cell lines.

To elucidate the effect of sustained Hes1 expression on ES cell differentiation, we used two independent lines of ES cells, R5 and R6, that have *Hes1* cDNA knocked-in into the Rosa26 locus (Hes1-sustained ES cells, Fig. 1A) (Kobayashi *et al.* 2009). These cells expressed Hes1 protein at a high level similar to the endogenous maximal level in a sustained manner (Fig. 1B,C) (Kobayashi *et al.* 2009). These cells expressed Oct3/4 protein and other ES cell markers and proliferated on feeder cells at similar levels to the parental wild-type ES cells (data not shown) (Kobayashi *et al.* 2009). Furthermore, these Hes1-sustained ES cells were able to form three germ layers in embryoid body (EB) and chimeric embryo formation assays (Kobayashi *et al.* 2009). Thus, both the self-renewal and the multipotential activities are not affected by sustained Hes1 expression.

**Figure 2:**
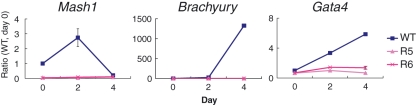
Kinetics of marker gene expression after the removal of LIF and feeder cells. After the removal of feeder cells, embryonic stem (ES) cells were cultured on gelatin-coated plate with LIF-removed ES cell medium (day 0). mRNA levels of marker genes, *Mash1*, *Brachyury* and *Gata4*, in the control (WT; blue) and Hes1-sustained ES cells (R5 and R6; red) were analyzed by quantitative real-time PCR. Each value was given in the ratio to the wild-type cells (WT) on day 0.

We next examined the differentiation of Hes1-sustained ES cells after the removal of both LIF and feeder cells, a condition known to induce all three germ layers (Rathjen & Rathjen 2001). We measured the expression kinetics of three germ layer markers, *Mash1* (neuroectodermal), *Brachyury* (early mesodermal) and *Gata4* (endodermal), by quantitative real-time PCR (Q-PCR). Expression of all marker genes was activated in the control cells within 4 days after withdrawal of LIF and feeder cells (WT, Fig. 2), but neither *Mash1*, *Brachyury* nor *Gata4* expression was significantly up-regulated in Hes1-sustained ES cells (R5 and R6, Fig. 2). We previously found that differentiation was delayed in EB formation derived from Hes1-sustained ES (R5 and R6) cells, although all three germ layers were eventually formed (Kobayashi *et al.* 2009). Thus, sustained Hes1 expression does not completely inhibit but just does delay ES cell differentiation.

### Inhibition of the neural fate choice by sustained Hes1 expression

**Figure 3:**
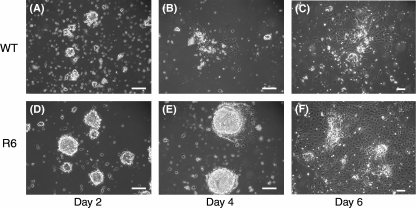
Comparison of the cell morphology and the growth under a neural differentiation condition. Phase contrast view of the wild-type (WT, upper panel) and Hes1-sustained embryonic stem cells (R6, lower panels). Cells were cultured in the N2B27 medium for 2, 4 and 6 days. Scale bar; 100 μm.

**Figure 4:**
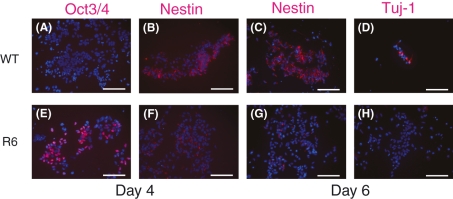
Comparison of marker protein expression under a neural differentiation condition. The control (WT, upper panels) and Hes1-sustained cells (R6, lower panels) cultured in N2B27 medium were analyzed on days 4 and 6 by immunocytochemistry using anti-Oct3/4, anti-Nestin and anti-Tuj-1 antibodies (red) with DAPI staining (blue). Scale bars, 100 μm.

In our previous study, we generated ES cells with the Venus sequence knocked-in into the Hes1 locus, so that Venus-Hes1 fusion protein was expressed from the endogenous *Hes1* promoter. We separated Hes1-high and Hes1-low ES cells by the Venus fluorescence and found that Hes1-high ES cells tended to differentiate into the mesodermal fate rather than the neural fate (Kobayashi *et al.* 2009), although past investigation has shown that activation of Notch signaling promotes neural differentiation (Lowell *et al.* 2006). We therefore examined the fate preference of Hes1-sustained ES cells under a neural differentiation condition (Ying & Smith 2003). Under this condition, the wild-type ES cells (WT) started changing morphology by day 4 (Fig. 3A,B) and had neural progenitor-like morphology by day 6 (Fig. 3C). Furthermore, these cells became negative for Oct3/4 expression (Fig. 4A) but positive for the neural progenitor marker Nestin on day 4 (Fig. 4B). On day 6, more cells expressed Nestin strongly (Fig. 4C), and subsets of cells expressed the neuronal marker βIII-tubulin (Tuj-1) (Fig. 4D). These results indicate that many wild-type ES cells efficiently differentiated into neural cells by day 6 under this condition. In contrast, Hes1*-*sustained ES cells continued to proliferate and enlarge their colonies until day 4 (Fig. 3D,E), but these colonies expanded with a flat non-neural morphology on day 6 (Fig. 3F). Many of these Hes1*-*sustained cells still expressed Oct3/4 on day 4 (Fig. 4E) but not on day 6 (data not shown), suggesting that ES cell differentiation is delayed by sustained Hes1 expression. Very few Hes1*-*sustained cells expressed Nestin on days 4 and 6 (Fig. 4F,G), and completely no cells expressed Tuj-1 (Fig. 4H). Thus, Hes1-sustained ES cells did not adopt the neural fate even under a neural differentiation condition. Because *Hes1* is known to inhibit neuronal differentiation from neural progenitor cells (Kageyama *et al.* 2007), the lack of Tuj-1^+^ neuron formation from Hes1-sustained ES cells was not surprising. However, the finding that even Nestin^+^ neural stem/progenitor cells were not well formed from Hes1*-*sustained ES cells was rather unexpected, because *Hes1* is highly expressed by neural stem/progenitor cells and is required for their maintenance (Kageyama *et al.* 2007). These results suggest that Hes1 has a different role in ES cells than in other stem/progenitor cells and that sustained Hes1 expression not only delays ES cell differentiation but also converts their differentiation into the non-neural fate.

### Preferential choice of the early mesodermal fate by sustained Hes1 expression

**Figure 5:**
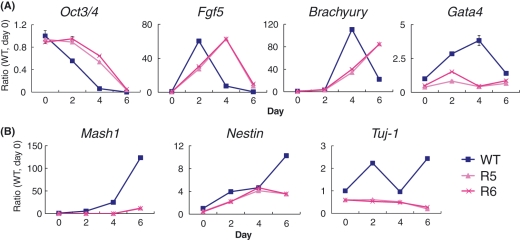
Kinetics of marker gene expression under a neural differentiation condition. mRNA levels of marker genes in the control (WT; blue) and Hes1-sustained embryonic stem cells (R5 and R6; red) under a neural differentiation condition were analyzed by quantitative real-time PCR. Each value was given in the ratio to the wild-type cells (WT) on day 0. (A) mRNA levels of marker genes, *Oct3/4*, *Fgf5*, *Brachyury* and *Gata4.* (B) mRNA levels of neural marker genes, *Mash1*, *Nestin* and *Tuj-1*.

Hes1-sustained ES cells did not differentiate into neural cells (Fig. 4F-H) but displayed a flat non-neural morphology on day 6 after induction of differentiation (Fig. 3F), suggesting that sustained Hes1 expression leads to non-neural cell differentiation with delayed timing. To elucidate which cell fate these Hes1*-*sustained ES cells tend to adopt, we quantified the mRNA levels of marker genes by Q-PCR on days 0, 2, 4 and 6 after neural induction. Consistent with the result of immunocytochemistry, decrease of *Oct3/4* expression was slower in Hes1*-*sustained (R5, R6) cells than in the wild-type cells (Fig. 5A). All cell lines showed transient up-regulation of *Fgf5*, an early marker of ES cell differentiation (Kunath *et al.* 2007), but the peak was delayed in Hes1*-*sustained (R5, R6) cells (Fig. 5A). The expression of *Mash1*, *Nestin* and *Tuj-1* was significantly repressed in Hes1*-*sustained (R5, R6) cells (Fig. 5B). In contrast, *Brachyury* was continuously up-regulated on days 4 and 6 in Hes1*-*sustained (R5, R6) cells, while *Gata4* was repressed during this period (Fig. 5A). *Goosecoid,* another early mesodermal marker gene (Liu *et al.* 2007), was relatively up-regulated in Hes1-sustained cells, but other mesodermal marker genes such as *Nkx-2.5* (cardiac lineage), *SCL* (hematopoietic lineage) and *Flk-1* (endothelial precursor) (Naito *et al.* 2006) were not significantly up-regulated even on day 6 in Hes1-sustained cells (data not shown). We also examined expression of the trophectodermal markers *Cdx2* and *Mash2* (Ivanova *et al.* 2006), but these genes were strongly suppressed in Hes1-sustained ES cells (data not shown). Thus, sustained Hes1 expression promoted the preference for the mesodermal rather than the neuroectodermal and trophectodermal fates, but mesodermal differentiation seemed to be halted at an early phase.

### Mesodermal differentiation of Hes1-sustained cells after down-regulation of Hes1

**Figure 6:**
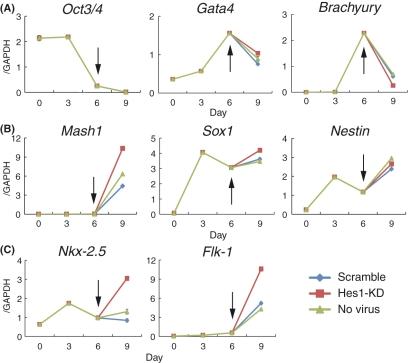
Hes1 knockdown promotes further mesodermal differentiation in Hes1-sustained cells. mRNA kinetics of marker genes, *Oct3/4*, *Gata4* and *Brachyury* (A), neural marker genes, *Mash1*, *Sox1* and *Nestin* (B), and mesodermal lineage marker genes, *Nkx-2.5* and *Flk-1* (C) were measured by quantitative real-time PCR. Arrows show the infection of lentivirus vectors carrying shRNA of scramble (blue) or Hes1 knockdown (Hes1-KD, red) into Hes1-sustained cells on day 6. Cells without virus infection were also analyzed (green).

The above results suggest that sustained Hes1 expression promotes the fate choice of mesodermal cells but inhibits further differentiation. Because Hes1 inhibits maturation of many cell types (Kageyama *et al.* 2007), we next examined whether Hes1 down-regulation promotes further differentiation of these cells. To this end, Hes1-sustained cells were subjected to Hes1 shRNA lentivirus vectors on day 6, when Oct3/4 was down-regulated, and were collected for mRNA quantification on day 9. As a control, Hes1-sustained cells with infection of no virus or control virus (scrambled shRNA sequence) were used. The knockdown efficiency of Hes1 shRNA lentivirus vectors was not so high (about 30% reduction), because the virus infection rate was low due to cell clumps. Nevertheless, we observed a significant effect on mesodermal differentiation in Hes1 knockdown cells. While early mesodermal markers (*Brachyury* and *Goosecoid*) were transiently up-regulated on day 6 and decreased on day 9 in both control and Hes1 knockdown cells (Fig. 6A and data not shown), the cardiac lineage marker *Nkx-2.5* and the endothelial marker *Flk-1* were significantly up-regulated in Hes1 knockdown cells compared with controls on day 9 (Fig. 6C). However, other mesodermal marker genes, such as *SCL* as well as *Oct3/4* and endodermal markers (*Gata4* and *Sox17),* were not significantly affected by Hes1 knockdown (Fig. 6A and data not shown). While *Mash1*, a direct target of Hes1, was slightly up-regulated, other neural genes were not significantly changed by Hes1 knockdown (Fig. 6B). These results suggest that sustained Hes1 expression halts mesodermal differentiation at an early phase, but that subsequent down-regulation of Hes1 allows further differentiation of some mesodermal lineages.

When Hes1 shRNA lentivirus vectors were infected with Hes1-sustained cells on day 4 after differentiation, *Nkx-2.5* and *Flk-1* were not up-regulated, but *Mash1* was significantly up-regulated on day 6 (data not shown). These results suggest that Hes1-sustained cells still maintain the potential of neuronal differentiation on day 4 after differentiation. This was probably because Oct3/4 was still expressed in these cells on day 4 (Fig. 4E). These results suggest that the irreversible commitment to the mesodermal fate occurred between days 4 and 6 after induction.

### Inhibition of Notch signaling by sustained Hes1 expression

**Figure 7:**
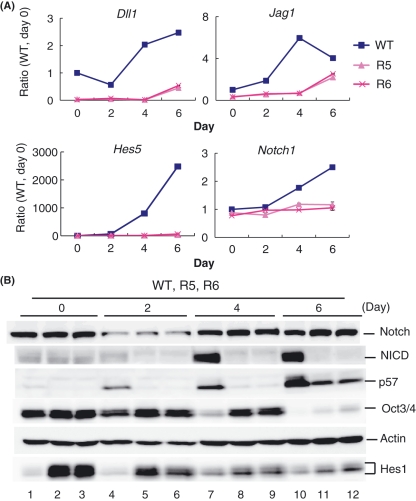
Hes1 regulates expression of Notch signaling and cell cycle factors during differentiation. (A) mRNA levels of Notch signaling genes, *Dll1*, *Jag1, Hes5* and *Notch1*. (B) Protein levels of Notch1 receptor, Notch intracellular domain (NICD), p57, Oct3/4 and Hes1 in the wild-type cells (WT; lanes 1, 4, 7 and 10) and in Hes1-sustained cells (R5; lanes 2, 5, 8 and 11, R6; lanes 3, 6, 9 and 12) under a neural differentiation condition.

Our results indicate that Hes1 and Notch signaling have opposite functions in ES cells, raising the possibility that Hes1 acts as an inhibitor rather than an effector of Notch signaling in these cells. We next tested this hypothesis. We quantified the mRNA levels of the Notch ligands *Dll1* and *Jag1* and a more faithful Notch effector gene, *Hes5* (because *Hes1*, another Notch effector, is up-regulated by other signaling molecules, such as LIF, BMP and FGF) (Nakayama *et al.* 2008; Kobayashi *et al.* 2009), by Q-PCR after neural induction. In the wild-type cells, *Dll1*, *Jag1*, *Notch1* receptor and *Hes5* expression were up-regulated on days 4 and 6 (Fig. 7A), suggesting that Notch signaling becomes active after neural induction. In contrast, all *Dll1*, *Jag1, Notch1* and *Hes5* expression was strongly repressed in Hes1*-*sustained (R5, R6) cells (Fig. 7A). Furthermore, our ChIP-chip analysis (chromatin immunoprecipitation followed by microarray analysis) revealed that Hes1 directly binds to *Dll1* and *Jag1* promoter regions in ES cells but not to *Notch1* or *Hes5* promoter regions (Kobayashi *et al.* 2009). These results together suggest that sustained Hes1 expression inhibits the activation of Notch signaling by directly repressing Notch ligand expression.

To obtain further evidence that activation of Notch signaling is inhibited by sustained Hes1 expression, we measured the levels of NICD, a processed active form of Notch1, by western blotting (Fig. 7B). NICD protein was detectable as faint bands in both the wild-type and Hes1*-*sustained ES (R5, R6) cells when these cells grew in self-renewal ES medium (Fig. 7B, lanes 1-3). After neural induction, the NICD level was slightly up-regulated on day 2 (Fig. 7B, lane 4) and became very high on days 4 and 6 in the wild-type cells (Fig. 7B, lanes 7 and 10). Thus, Notch signaling is very weak in self-renewing mouse ES cells as in human ES cells (Noggle *et al.* 2006) but is strongly activated during neural differentiation. In contrast, NICD was kept mostly undetectable in *Hes1-*sustained (R5, R6) cells after neural induction (Fig. 7B, lanes 5, 6, 8, 9, 11 and 12). Notch1 receptor expression levels were slightly higher in *Hes1-*sustained cells (Fig. 7B, lanes 8, 9, 11 and 12) than in the wild-type cells (Fig. 7B, lanes 7 and 10), suggesting that this increase is attributable to blockade of NICD formation in Hes1-sustained cells. These results indicate that sustained Hes1 expression leads to inactivation of Notch signaling. In these cells, Oct3/4 expression was maintained until day 4 (Fig. 7B, lanes 5, 6, 8 and 9), which agreed well with the results of immunocytochemistry (Fig. 4E) and Q-PCR (Fig. 5A).

In the wild-type cells, the Hes1 level was initially low on days 0 and 2 (Fig. 7B, lanes 1 and 4) but up-regulated on days 4 and 6, when Notch signaling became active (Fig. 7B, lanes 7 and 10). It is likely that Notch activation contributes to a higher level of Hes1 expression in the wild-type cells at a later phase, although the mechanism of such switching of Notch dependency remains to be determined. In Hes1-sustained ES cells, Hes1 was expressed at high levels, but the expression gradually decreased after neural induction (Fig. 7B, lanes 2, 3, 5, 6, 8, 9, 11 and 12), suggesting that such decreased levels of Hes1 are not sufficient to inhibit differentiation of these ES cells.

We also examined the expression of p57, a G1/S cyclin-dependent kinase inhibitor, which was identified as an indirect downstream gene for Hes1 in ES cells (Kobayashi *et al.* 2009). The expression of p57 increased during differentiation in the wild-type cells (Fig. 7B, lanes 4, 7 and 10), but the increase was significantly delayed in Hes1-sustained (R5, R6) cells (Fig. 7B, lanes 5, 6, 8, 9, 11 and 12), suggesting that sustained Hes1 expression promotes proliferation and delays the cell cycle exit of these cells. These results together indicate that sustained Hes1 expression suppresses Notch signaling, delays the cell cycle exit and inhibits the neural fate choice.

## Discussion

### Hes1 regulates the fate choice in ES cell differentiation by controlling Notch signaling

**Figure 8:**
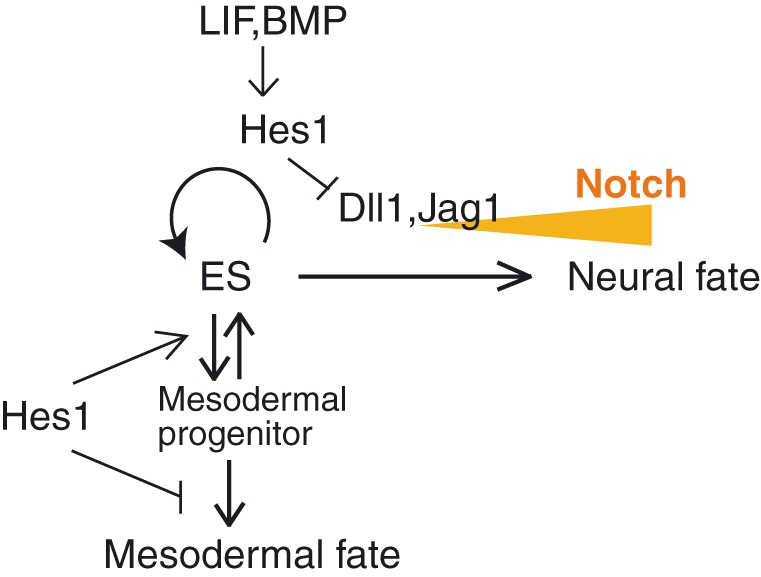
Proposed model. In embryonic stem (ES) cells, Hes1 expression is controlled by LIF and BMP. Hes1 suppresses Notch signaling by repressing *Dll1* and *Jag1* expression and thereby inhibiting neural differentiation. Sustained Hes1 expression leads ES cells to the mesodermal differentiation but inhibits the following commitment probably at the mesodermal stem/progenitor state.

In our recent study, we have shown that Hes1 expression oscillates in ES cells and that this oscillation contributes to diversity in the fate choice of ES cell differentiation (Kobayashi *et al.* 2009). Interestingly, Hes1 is not essential for maintenance of the stem cell state of ES cells, but Hes1 levels at the time of induction of differentiation are critical for the fate choice: Hes1-high cells are prone to the mesodermal fate and Hes1-low cells are prone to the neuroectodermal fate (Kobayashi *et al.* 2009). The mechanism of how Hes1 regulates the fate choice in ES cell differentiation remained to be analyzed. Previous studies revealed that activation and inactivation of Notch signaling in ES cells lead to preferential fate determination of the neuroectoderm and the mesodermal cardiomyocyte, respectively (Lowell *et al.* 2006; Schroeder *et al.* 2003; Nemir *et al.* 2006; Jang *et al.* 2008). In this study, we found that ES cells with sustained Hes1 expression tend to differentiate into *Brachyury*-positive mesodermal lineage. Thus, the phenotypes of sustained Hes1 expression and those of Notch inactivation are similar to each other, and therefore one of the mechanisms for Hes1-induced mesodermal fate choice could be the suppression of Notch signaling. We found indeed that sustained Hes1 expression in ES cells suppresses Notch signaling (repressing expression of the Notch ligands *Dll1* and *Jag1*, inhibiting formation of NICD, and repressing the expression of a faithful Notch effector, *Hes5*). In contrast, our recent study has found that *Hes1*-null ES cells, which express higher levels of *Dll1* and *Jag1*, tend to differentiate into neural cells (Kobayashi *et al.* 2009). Thus, Hes1 regulates the fate choice in ES cell differentiation by controlling Notch signaling activity (Fig. 8).

### Hes1 functions as a regulator and an effector of Notch signaling

In the wild-type ES cells, Hes1 expression was up-regulated under a neural differentiation condition on days 4 and 6, when Notch signaling was very active. We previously established a live imaging system for *Hes1* expression in ES cells by using Hes1 promoter-driven destabilized luciferase as a reporter (Masamizu *et al.* 2006; Kobayashi *et al.* 2009). In this assay, we found that *Hes1* expression in ES cells was transiently down-regulated upon induction of neural differentiation, but that *Hes1* oscillations resumed 2 days later (our unpublished data). We speculate that upon induction of differentiation, new synthesis of Hes1 protein is blocked transiently, and that the Hes1 protein levels could be fixed for a while, leading to distinct activities of Notch signaling and adoption of distinct types of stem/progenitor cells, where *Hes1* oscillations resume. This hypothesis has not yet been tested, because live imaging of Hes1 protein expression over time is technically difficult. It is likely that some cells lose Hes1 protein upon induction of neural differentiation, because new synthesis of Hes1 protein is transiently blocked after removal of both LIF and BMP, and that in these cells the reduction in the Hes1 level is a trigger for induction of Notch ligand expression and activation of Notch signaling. After activation of Notch signaling, Hes1 expression seems to be induced as an effector, just like a more faithful Notch effector, *Hes5*. Thus, we speculate that Hes1 has distinct functions, as an inhibitor and an effector of Notch signaling, depending on stages (Fig. 8). In Hes1-sustained ES cells, Notch signaling is kept inactive, and the function as a regulator (inhibitor) of Notch signaling seems to be continuously dominant.

### Different functions of Hes1 in cell differentiation

The result that Hes1 expression levels at the time of induction of differentiation affect the cell fate choice of ES cells is rather unexpected, because Hes1 is expressed by neural, mesodermal and endodermal stem/progenitor cells (Sasai *et al.* 1992; Jensen *et al.* 2000). Hes1 expression itself can be compatible with the cell fates of all three germ layers, or rather, it is required for maintenance of stem/progenitor cells of all three germ layers. For example, Hes1 plays an essential role in maintenance of neural stem/progenitor cells, and without Hes1, these cells prematurely differentiate into neurons (Ishibashi *et al.* 1995; Shimojo *et al.* 2008). Apparently, Hes1-sustained ES cells preferentially adopted the mesodermal fate upon induction of differentiation but were halted at an early phase, probably at the mesodermal stem/progenitor state (Fig. 8). Down-regulation of Hes1 promoted further mesodermal differentiation, suggesting that Hes1 promotes adoption of the mesodermal fate but inhibits further differentiation. Thus, the Hes1 functions seem to be different between ES cells and somatic stem/progenitor cells, and further analysis is required to clarify this issue.

Hes1-sustained ES cells were delayed in differentiation for several days, although the cells eventually started differentiation. We found that Oct3/4 expression was well maintained until day 4 in these cells even after LIF and BMP were removed. On day 6 after differentiation induction, Oct3/4 expression was lost in Hes1-sustained cells. At this stage, Hes1 expression also decreased in these cells (Fig. 7B, lanes 11 and 12). Thus, it is not clear whether the loss of Oct3/4 expression was because of the decrease in the Hes1 expression level or of the insufficiency of Hes1 for maintenance of ES cells for a longer time. To answer this question, it will be required to test whether higher levels of Hes1 can maintain ES cells for a longer time without LIF and BMP. The mechanism of how Hes1-sustained ES cells keep Oct3/4 expression also remains to be determined. Because Hes1 promotes Jak-induced activation of Stat signaling (Kamakura *et al.* 2004), sustained Hes1 expression could substitute for LIF signaling to some extent.

Our results in this study raise important implications for the application of ES cells to regenerative medicine. ES cells have multipotential activities, but currently it is difficult to control the fate choice of ES cells: ES cells tend to asynchronously differentiate into diverse cell types in a rather chaotic manner. Our data indicate that various Hes1 expression levels are involved in such diversity of ES cells in differentiation responses. Thus, we suggest that manipulation of Hes1 expression levels is one of the methods to overcome the problems of ES cell regulation.

## Experimental procedures

### ES cell lines and culture condition

TT2 ES cell line was used for this study. Genetic manipulation of *Hes1*-sustained ES cells was described previously (Kobayashi *et al.* 2009). ES cells were maintained in DMEM medium supplemented with 15% fetal calf serum (FCS), l-glutamine, non-essential amino acids, sodium pyruvate, 2-mercaptoethanol and 1000 U/mL LIF on a feeder cell layer of mouse embryonic fibroblasts. Before the differentiation assay, feeder cells were removed twice by floating incubation on gelatin-coated plate for 30 min after trypsinization. Neural differentiation was induced in N2B27 medium, as previously described (Ying & Smith 2003).

### Lentivirus vectors for Hes1 knockdown

pCSII vector (Miyoshi 2004), carrying shRNA for Hes1 knockdown or scrambled sequence under the 7SK promoter (Kobayashi *et al.* 2009) and GFP gene under the EF promoter, was used for virus production.

### Real-time PCR and immunostaining

Quantification by real-time PCR (Q-PCR) and western blotting was carried out, as previously described (Yoshiura *et al.* 2007; Kobayashi & Ito 1999). For all real-time PCR analysis, a standard curve was drawn for each primer set using mixtures of cDNA samples. Quantified values of RNA were normalized with those of GAPDH and shown by the average with an error bar of two independent experiments. Primer sequences were described previously (Kobayashi *et al.* 2009). For immunocytochemistry, cells were fixed with 4% paraformaldehyde (PFA) on ice, blocked with 0.1% Triton-2% skim milk in phosphate-buffered saline (PBS) and stained with specific antibodies.

### Antibodies

The following antibodies were used for western blotting: rabbit anti-Hes1 antibody (gift from Dr. Tetsuo Sudo), goat anti-Oct3/4 (N-19) and anti-p57 (M-20) antibodies (Santa Cruz Biotechnology), rabbit anti-actin antibody (SIGMA, A2066) and rabbit anti-cleaved Notch1 (NICD) antibody (Cell Signaling Technology). For immunocytochemistry, the following antibodies were used: rabbit anti-Tuj-1 antibody (Covance), mouse anti-Nestin, anti-Oct3/4 antibodies (BD Pharmingen) and rabbit anti-Hes1 antibody (Kobayashi *et al.* 2009). The following secondary antibodies were used for fluorescent labeling: Alexa488-conjugated anti-rabbit and anti-mouse antibodies, and Alexa594-conjugated anti-rabbit and anti-mouse antibodies (Molecular Probes).
